# The Spectrum of MRI and Electrographic Findings in Pediatric Patients With Seizures: A Retrospective Tertiary Care Center Study

**DOI:** 10.7759/cureus.35851

**Published:** 2023-03-07

**Authors:** Reem A Alyoubi, Razan K Daghistani, Afaf M Albogmi, Taif A Alshahrany, Alzahraa B AlAhmed, Tala A Fayoumi, Fahad A Alotibi, Anas S Alyazidi

**Affiliations:** 1 Department of Pediatrics, King Abdulaziz University Hospital, Jeddah, SAU; 2 Department of Pediatrics, Faculty of Medicine, King Abdulaziz University, Jeddah, SAU; 3 Department of Radiology, Faculty of Medicine, King Abdulaziz University, Jeddah, SAU; 4 Department of Medicine, King Abdulaziz University Hospital, Jeddah, SAU; 5 Faculty of Medicine, King Abdulaziz University, Jeddah, SAU

**Keywords:** eeg, mri, saudi arabia, neuroimaging, seizures

## Abstract

Background

Seizures constitute a serious public health concern, especially in pediatric patients. They are among the most prevalent medical issues affecting children. Magnetic resonance imaging (MRI) is a widely used imaging modality to evaluate, assess, and follow up on brain abnormalities.

Objectives

The purpose of the study is to explore the clinical findings of pediatric patients with seizures and their associated findings on MRI examinations.

Material and methods

A retrospective cohort study was conducted between 2021 and 2022 at King Abdulaziz University Hospital (KAUH). A total of 171 pediatric patients (ages 1-14 years old) who had seizures and underwent brain MRI examinations were included in the study. The mean age of the group was two years.

Results

Focal seizures represented the majority of seizure types in 62 (60.2%) patients compared to 42 patients who presented with generalized seizures and 67 patients who presented with normal findings based on magnetic resonance imaging (MRI) analysis. The most common finding in imaging was the presence of developmental anomalies, which were found in 31 (18.5%) patients. This was followed by hypoxic-ischemic injury in 12 patients, vascular abnormalities in 10 patients, inherited metabolic disorders in nine patients, and infection-related findings in six patients.

Conclusion

MRI has an invaluable role in managing pediatric patients with seizures. Accurate diagnosis of patients is an essential step for delivering proper care to patients. MRI is considered the main imaging modality to establish a correct diagnosis and thereby improve prognosis, and electroencephalogram (EEG) should be taken into account during standard neurodiagnostic testing.

## Introduction

Seizure is a condition of the cerebral cortex in which symptoms are caused by an abnormal, spontaneous, and excessive discharge of neurons [1]. It represents a chronic and serious neurological problem and a major public health concern [2,3]. Children diagnosed with seizures encounter different forms of challenges [4]. When a patient experiences a seizure, there are important etiologies to consider, which include tumors, traumatic brain injury, hemorrhage, infection, and hereditary disease, which are important to exclude before making a diagnosis of epilepsy [1]. Electroencephalography and neuroimaging are essential in the initial assessment of patients with seizures as they can identify the cause of seizure attacks [5]. These modalities are advantageous given that they are noninvasive and do not expose the patients to ionizing radiation [6]. Magnetic resonance imaging (MRI) is the complementary modality of choice to electroencephalogram (EEG) due to its ability to depict neuroanatomy and its high sensitivity [6]. It plays a fundamental role in demonstrating the morphology, distribution, and extent of cortical involvement [7]. Identification of the underlying structural lesions causing seizure is critical and requires a complete clinical and radiological evaluation [8,9]. MRI, in addition to establishing a diagnosis, has a role in evaluating pediatric patients with seizures, aiding in planning treatment, and establishing the patient’s prognosis [6]. To the extent of our knowledge, there are no available data in Saudi Arabia on the role of MRI in evaluating clinical findings for pediatric patients with seizures. On this note, this exploratory study aims to assess the role of MRI in pediatric patients with seizures and analyze the spectrum of findings associated with various etiologies.

## Materials and methods

Study design and setting

Following the Strengthening the Reporting of Observational Studies in Epidemiology (STROBE) guidelines for retrospective studies, this record review study was performed using the electronic “Phoenix” hospital record system. Data were obtained by reviewing medical reports of pediatric patients who presented with provoked or unprovoked seizures from January 2021 to December 2022. Patients were identified through Pediatric Neurology and Neuroradiology Department databases at King Abdulaziz University Hospital (KAUH), a tertiary care center in Jeddah, Saudi Arabia. Ethical approval was obtained from the Unit of Biomedical Ethics at the Faculty of Medicine, King Abdulaziz University with reference number 209/21.

Study population

All pediatric patients of ages 1-14 years old who presented with seizures and those who underwent brain MRI were included in the study, while all patients less than one-year-old were excluded.

Data collection instruments

The collected data included the patient’s demographic characteristics (age, gender, nationality, place of origin, diagnosis date, admission site, and previous history of epilepsy), seizure occurrence (type, duration, character, and frequency of epileptic attacks), cause of admission (prolonged febrile seizure, central nervous system (CNS) infection, electrolyte imbalance, hemorrhagic stroke, and neoplastic), history (cerebral palsy, metabolic disorder, epilepsy, and neoplasm), and MRI findings. Additional EEG findings (performed, not performed, and performed before MRI) were also obtained.

Data analysis

Data entry was performed using Microsoft Excel version 16.37 (Microsoft Corporation, Redmond, WA, USA), and Statistical Package for the Social Sciences (SPSS) version 21 (IBM SPSS Statistics, Armonk, NY, USA) was used for statistical analysis. Means and standard deviations were calculated to describe continuous variables, while numbers and percentages were used for categorical variables. Student t-test and chi-square test were used to evaluate the differences between continuous and categorical variables, respectively. Statistical significance was achieved if the p-value was <0.05, with a 95% confidence interval.

## Results

A total of 171 pediatric cases were collected after fulfilling the selection criteria of the study. Among them, 76 (44.5%) were female cases and 95 (55.5%) were male cases. The age range was from one to 14 years old. The demographic characteristics of the enrolled cases were as follows. Sixty-eight patients presented with the generalized type of seizure, while 103 presented with focal seizures based on clinical records. The predominant seizure characteristic among the cases was the intermittent seizure pattern, which manifested in 105 (61%) patients, compared to the continuous pattern, which manifested in 66 (38%) patients. The causes of the seizures were as follows: unknown etiology in 67 (39%) patients, prolonged febrile seizure in 40 (23%) patients, electrolyte imbalance in 37 (21%) patients, central nervous system (CNS) infection in 14 (8%) patients, a trauma in seven (4%) patients, hemorrhagic stroke in five (3%) patients, and a single patient with neoplasm (0.6%). Other demographic data were presented in Table 1.

**Table 1:** Demographic characteristics of the included cases

Demographic characteristics	Number (%)
Gender	
Female	76 (44.5)
Male	95 (55.5)
Nationality	
Non-Saudi	80 (46.8)
Saudi	91 (53.2)
Origin	
Jeddah	58 (33.9)
Makkah	2 (1.2)
Other	111 (64.9)
Admission route	
Emergency department	151 (88.3)
Intensive care unit	20 (11.7)
Types of seizure	
Generalized	68 (39.8)
Focal (partial)	103 (60.2)
Seizure character	
Continuous	66 (38.6)
Intermittent	105 (61.4)
Causes	
Prolonged febrile seizure	40 (23.4)
Central nervous system infections	14 (8.2)
Electrolyte imbalance	37 (21.6)
Hemorrhagic stroke	5 (3)
Neoplasm	1 (0.6)
Trauma	7 (4.1)

Furthermore, the type of seizure in each gender was compared in Table 2 among different causes of seizures. The results yielded indicated that male patients with generalized seizures due to prolonged febrile seizures were more prevalent in comparison to female patients. However, there are two major types of seizures: generalized and focal. In the focal form of seizure, the frequency of occurrence was equal in both genders. CNS infections were more prevalent among males in both the generalized and partial forms of seizures with no female reporting having a generalized seizure caused by CNS infection. Male patients were more likely to experience seizures as a result of electrolyte imbalance than their female counterparts. Hemorrhagic strokes were likely to cause generalized seizures in males, but not in females, but equally caused focal seizures among both genders. A single neoplastic case was reported in a female patient, which caused her a generalized form of seizure. Traumas were likely to cause seizures in a more prevalent manner in male patients than in female patients in both seizure forms. Detailed data were presented in Table 2.

**Table 2:** Type of seizure among both genders in relation to the seizure cause

	Type of seizure			
	Generalized		Focal	
	Female	Male	Female	Male
Prolonged febrile seizure	7	11	11	11
Central nervous system infection	0	4	4	6
Electrolyte imbalance	5	10	10	12
Hemorrhagic stroke	0	3	1	1
Neoplastic	1	0	0	0
Trauma	0	1	2	4

**Figure 1:**
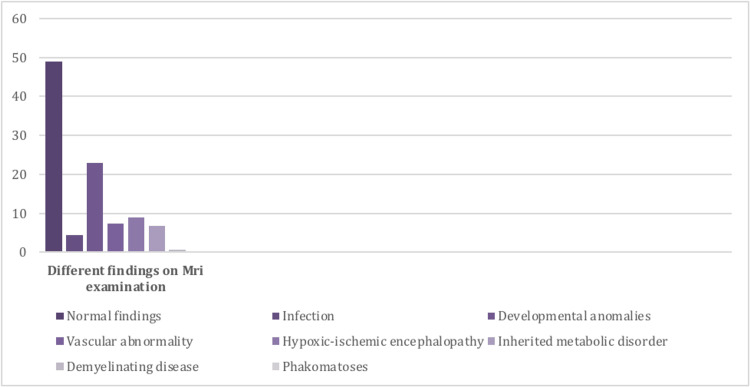
Different findings on magnetic resonance imaging examination

According to magnetic resonance imaging (MRI) analysis, 67 (39.81%) patients presented with normal findings. The abnormal MRI findings found were related to the following etiologies: developmental anomalies, vascular abnormality, hypoxic-ischemic injury, infection, inherited metabolic disorder, and demyelinating disease (Figure 1).

**Figure 2:**
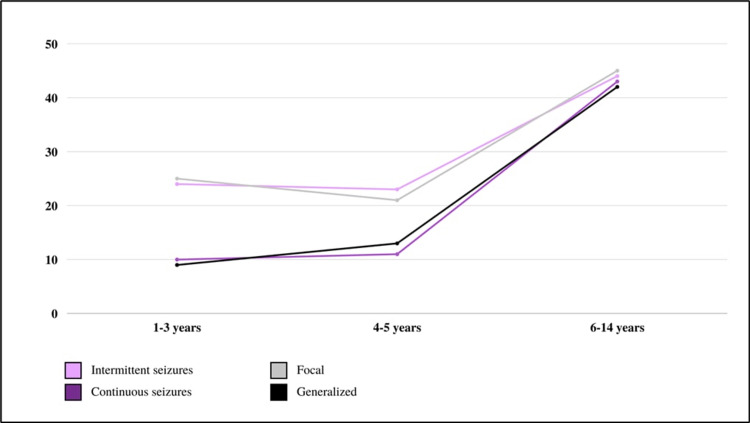
Seizure characteristics according to different age groups

After grouping the patients according to their ages, 16 patients were 1-12 years old, 34 patients were 1-3 years old, 34 patients were 4-5 years, and 87 patients were 6-14 years old. A linear chart in Figure 2 presents the characteristics of the seizure and its type according to age groups.

Finally, comparing abnormal EEG findings with abnormal MRI findings using the chi-square test, among the 26 patients who presented with developmental anomalies, only 18.5% had abnormal EEG findings. Furthermore, among patients with abnormal vascular findings, 12.5% presented with abnormal EEG. Also, 45.5% of patients with anoxia and/or hypoxic-ischemic injury presented with abnormal EEG results, and around 25% of patients with signs of inherited metabolic disorder presented with abnormal EEG results. Moreover, 11.1% of patients with incidental findings presented with abnormal EEG. Detailed data are presented in Table 3.

**Table 3:** Chi-square test for relevant seizure findings using magnetic resonance imaging compared to positive electroencephalogram findings

Abnormal electroencephalogram findings			Relevant findings related to seizure using magnetic resonance imaging
p-value	Positive findings	Number	
0.562	18.5%	26	Development anomalies
0.622	12.5%	30	Vascular
0.070	45.5%	26	Anoxia and hypoxic-ischemic encephalopathy
0.792	16.7%	30	Infection-related findings
0.874	25%	29	Inherited metabolic disorder
N/A	0%	30	Demyelination disease
0.343	11.1%	29	Incidental findings (e.g., cyst, not related to the seizure)

## Discussion

Seizures are potentially dangerous medical conditions. This paroxysmal event requires a clear explanation of the seizure prognosis and management by the patients, their families, and healthcare workers. Nearly 10% of people experience at least one form of seizure throughout their life [10]. The most common form of seizures in the pediatric population is febrile seizures, which affect 2%-5% of children and are a cause of great concern for children’s parents [11]. Seizures are associated with a significant physical burden and emotional burden that requires continuous medical care, especially for pediatric patients.

In our study, we highlighted MRI and EEG findings in patients with seizures. MRI was chosen due to its critical and essential role in the medical management of patients with seizures. MRI holds significant etiological diagnostic abilities [12]. The gender characteristics of the present study were similar to published literature that revealed male predominance [13]. Focal seizures were very common in the present study population, which was consistent with prior investigations [14]. In contrast, other studies observed a wider prevalence of generalized seizures among children with epilepsy [15]. Furthermore, patients with intermittent seizure patterns were roughly double the number of patients with continuous seizures [15]. Regarding seizure etiology, febrile seizures were the most frequently reported and represented in about 23.4% of the patients included in this study. Similar findings were observed in the literature [16]. Febrile seizures are benign self-limiting forms of seizures but could be worrying to most parents. They represent one of the most common causes of emergency room visits [11]. Given the benign nature of this condition, it was expected to be the main cause in children, especially in those from six months to five years as reported in other studies [17-19]. The findings of the present study reemphasized prior studies that have investigated febrile seizures most frequently [18,20].

Febrile seizures were more common in male patients. Other important etiologies include CNS infections and electrolyte imbalance. The latter, however, represents the second most commonly reported etiology. We employ MRI as part of neuroimaging in the early stages of epilepsy because it helps in identifying the causes and guides therapeutic options [21]. In a recent study, 40% of the patients had abnormal MRI findings in children with epilepsy, most of them having intact brains on MRI. The present study is comparable with a study done in the United States in which MRI revealed abnormalities in 43% of patients [22]. However, in the current study, all children who presented with seizures were included, which could be the most appropriate interpretation for the cause of the increased rate of abnormal MRI findings. This study further showed that developmental anomalies were the most common abnormal MRI findings in our pediatrics department, which was not a common find in published literature. This may be due to the high rate of consanguineous marriages in Saudi Arabia. The rate of consanguineous marriages was found to be up to 51.3% [23] in previous investigations and up to 39.8% in more recent studies [24]. Another study, however, concluded that CNS infection is the main etiology in pediatric patients with seizures. This difference is attributed to the fact that referral centers in India usually manifest a high number of cases of infection [25-27]. Nonetheless, other pathological findings that included developmental anomalies (18.5%), vascular abnormalities (12.5%), hypoxic-ischemic injury (45%), CNS infection (16.7%), inherited metabolic disorder (25%), and incidental findings (11%) were reported at different rates. In patients with positive family history, abnormal physical neurological examinations, or other clinical suspicions that include dysmorphic features and congenital anomalies, it is recommended to perform an MRI examination even if the EEG examination was normal. According to the literature, MRI is one of the integral modalities in evaluating seizure etiology [28].

Limitations of the study

Although this study showed great importance in detecting seizure etiologies, our study experienced some limitations. The study was limited by its small sample size, the lack of standardized terminology used to describe EEG, and the lack of previous studies nationwide to compare our findings. Therefore, verification with a larger and more distributed sample is recommended to avoid selection bias.

## Conclusions

The present study demonstrates the invaluable role of MRI in managing pediatric patients with seizures and reveals the spectrum of MRI findings associated with various causes among pediatric patients in Saudi Arabia. MRI has been shown to play an important role in the management of pediatric seizure patients along with other modalities that include the use of EEG. It should be able to aid caregivers in reaching an accurate diagnosis, which is essential to decide the necessary care for their patients. MRI is considered the main imaging modality to establish a correct diagnosis and hence help in achieving a good prognosis. Prolonged febrile seizures have been shown to be the most common cause of seizures according to the included sample.
